# Decoding Transdiagnostic Subtypes in Emotional Disorders

**DOI:** 10.1002/cns.71122

**Published:** 2026-09-01

**Authors:** Yun Wu, Chun Wang, Changjun Teng, Yan Lu, Chengbin Guan, Yuan Zhong

**Affiliations:** ^1^ School of Psychology Nanjing Normal University Nanjing China; ^2^ Taizhou Fifth People's Hospital, Jiangsu Taizhou Jiangsu China; ^3^ Adolescent Education and Intelligence Support Lab of Nanjing Normal University, Laboratory of Philosophy and Social Sciences at Universities in Jiangsu Province Nanjing China; ^4^ Department of Psychiatry The Affiliated Brain Hospital of Nanjing Medical University Nanjing Jiangsu China; ^5^ Department of Medical Psychology The Affiliated Brain Hospital of Nanjing Medical University Nanjing Jiangsu China; ^6^ Early Intervention Unit, Department of Psychiatry The Affiliated Brain Hospital of Nanjing Medical University Nanjing Jiangsu China

**Keywords:** emotional disorders, functional connectivity, nonnegative matrix factorization clustering, resting‐state fMRI, transdiagnostic

## Abstract

**Background:**

Emotional disorders (EDs), including anxiety disorders, major depression (MDD), and post‐traumatic stress disorder (PTSD), show overlapping symptoms and neurobiological heterogeneity, limiting current diagnostic frameworks. Transdiagnostic biomarkers are critical for precision psychiatry.

**Methods:**

Resting‐state functional connectivity (FC) from 261 ED patients and 201 healthy controls (HCs) was decomposed via nonnegative matrix factorization (NMF). Differential FC features defined latent disease factors, classifying patients into subtypes with distinct network/structural profiles.

**Results:**

The analysis identified five representative functional networks (RFNs) underlying the heterogeneity of emotional disorders. RFN1 reflected connectivity between sensorimotor and dorsal attention networks, while RFN2 was defined by theory‐of‐mind‐related connectivity. RFN3, characterized by thalamocortical connections, was specifically linked to somatic sensation processing. RFN4 involved connectivity within the default mode network, and RFN5 captured multi‐network‐subcortical integration. These RFNs delineated five subtypes with distinct clinical profiles: ST1 (mixed anxiety/depression) exhibited RFN1‐driven somatic vigilance and comorbid depressive symptoms; ST2 (mild symptoms) demonstrated resilience associated with RFN2; ST3 (somatic anxiety‐dominant) showed RFN3‐related elevations in somatic anxiety and panic symptoms; ST4 (anxiety‐centric) was marked by RFN4‐associated self‐referential anxiety; and ST5 represented a depression‐pure subgroup mapped to RFN5.

**Conclusion:**

This study proposes a neurobiologically informed stratification for EDs, revealing transdiagnostic subtypes with distinct network signatures and clinical implications. The findings bridge symptom heterogeneity to circuit‐level dysfunction, offering potential biomarkers for personalized interventions and advocating for transdiagnostic approaches in psychiatric research.

## Graphical abstract

Using nonnegative matrix factorization (NMF) clustering, our transdiagnostic study identified five representative functional networks (RFNs) that capture the heterogeneity of emotional disorders. These RFNs delineated five clinically distinct subtypes, each with unique diagnostic proportions, clinical symptomatology, and brain functional and structural patterns. This multidimensional taxonomy bridges the gap between behavioral symptoms and underlying neural circuit abnormalities, offering a systems‐level framework for understanding emotional disorders.

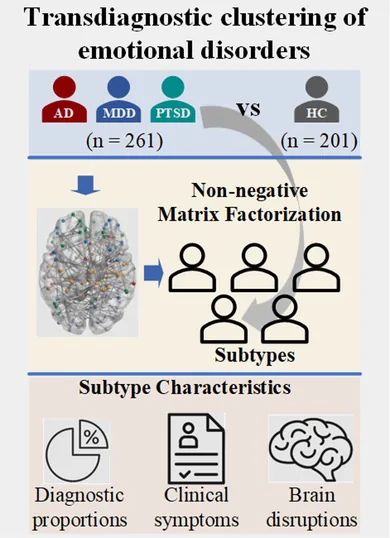

## 1 Introduction

Emotion‐related psychiatric disorders (emotional disorders, EDs), including anxiety disorders (ADs), major depressive disorder (MDD), and post‐traumatic stress disorder (PTSD), are characterized by dysregulated emotional processing and mood disturbances. While these disorders exhibit distinct diagnostic criteria, significant symptom overlap exists—such as shared negative emotional states, sleep disturbances, and attentional deficits [1, 2, 3]. Epidemiological studies further highlight their frequent comorbidity, with patients experiencing multiple EDs often presenting more severe symptoms, poorer functional outcomes, and reduced treatment responsiveness [4, 5, 6, 7]. Despite this clinical complexity, current diagnostic frameworks predominantly focus on single‐axis classifications, emphasizing disorder‐specific features rather than transdiagnostic mechanisms.

Phenomenology‐based diagnostic classifications fail to capture the neurobiological essence of psychiatric disorders, resulting in limited therapeutic guidance [8]. While neuroimaging has been widely adopted to identify disorder‐specific biomarkers, often conceptualized as distinct “lesions” [9], studies increasingly reveal shared structural and functional abnormalities across psychiatric conditions. This observation has spurred transdiagnostic research, such as investigations into common and distinct network‐level anomalies in bipolar disorder (BD) and MDD [10], as well as comparisons of transdiagnostic factors (e.g., distress, anxiety arousal, trauma) across AD, BD, and MDD [11]. These efforts aim to integrate fragmented symptom‐based perspectives into multidimensional frameworks. For frequently comorbid emotional disorders, a unified approach is needed to quantify the relationship between symptom combinations and diagnostic boundaries. However, limited sample sizes and research priorities have hindered comprehensive studies simultaneously addressing ADs, MDD, and PTSD.

Transdiagnostic meta‐analyses of ADs and MDD demonstrate shared gray matter volume (GMV) reductions in the orbitofrontal cortex (OFC), striatum, and limbic regions [12], alongside functional alterations in OFC and limbic regions [13]. To date, only two meta‐analyses have simultaneously included neuroimaging data for ADs, MDD, and PTSD. Serra‐Blasco et al. [14] identified a common GMV reduction in the left mid‐cingulate cortex across these three disorders [14], while Janiri et al. [15] analyzed task‐based functional magnetic resonance imaging (fMRI) findings across ADs, MDD, PTSD, and BD, revealing hypoactivation in salience‐processing regions and hyperactivation in emotion generation/expression regions [15].

Resting‐state functional connectivity (FC), reflecting intrinsic network dynamics, offers an objective biomarker sensitive to both behavioral symptoms and neuropathology. Nonnegative matrix factorization (NMF), a dimensionality reduction technique widely applied in neuroimaging [16, 17], enables decomposition of high‐dimensional FC data into latent disease factors. This approach facilitates subtype identification based on individualized factor loadings, providing a path toward precision psychiatry. While NMF has been utilized in studies of Alzheimer's disease and MDD, its application to transdiagnostic ED cohorts remains underexplored.

**FIGURE 1:**
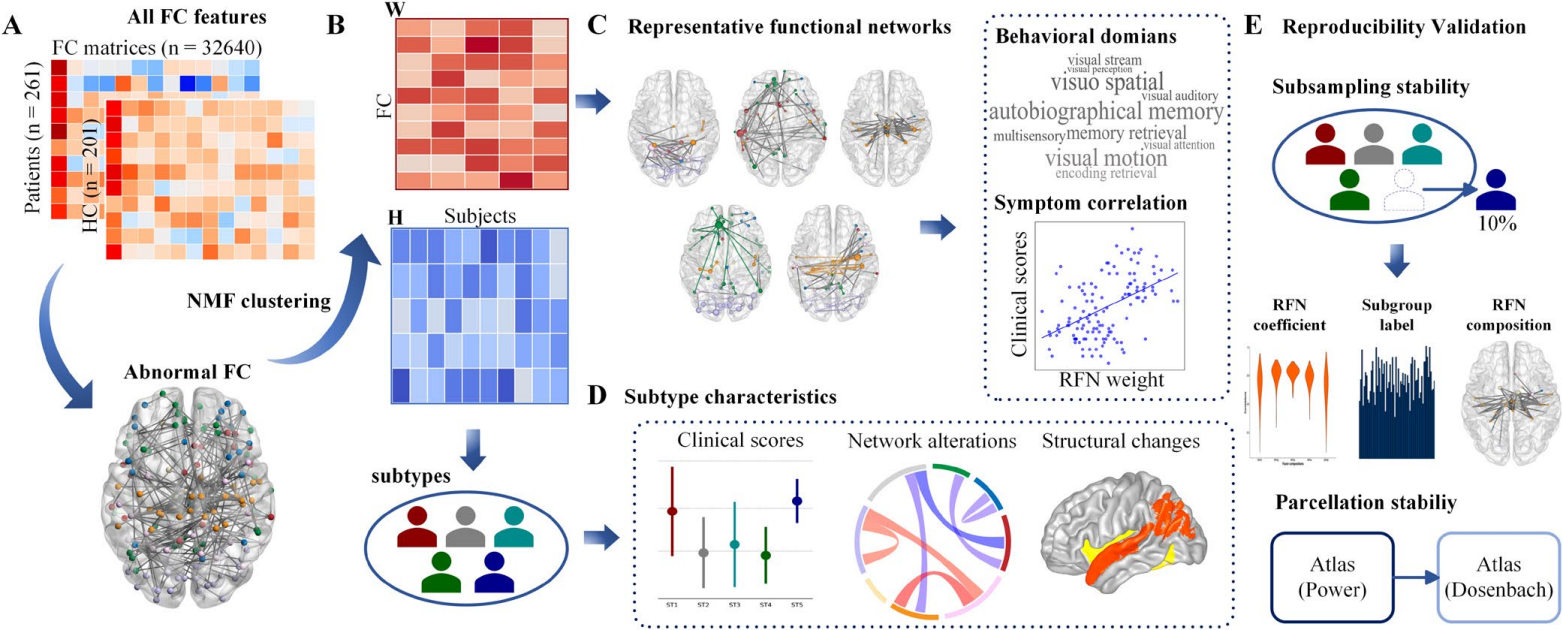
Schematic of the clustering analysis pipeline. (A) Functional connectivity (FC) features were derived from group‐level differences in Power‐264 atlas‐based FCs between patients with emotional disorders (ED) and healthy controls (HCs); (B) Nonnegative matrix factorization (NMF) clustered ED patients' FCs into five representative functional networks (RFNs), decomposing the original matrix into two components: W (FC × RFN) reflecting FC contributions and H (RFN × subject) encoding individual weights. Subjects were classified into five subtypes based on maximum H weights. Darker hues in W and H matrices indicate stronger FC contributions or subtype‐specific loadings; (C) RFN‐specific analyses included behavioral domain characterization and symptom correlation mapping; (D) demographic and symptomatic characteristics of the five identified subgroups, and specific changes in brain network and brain structural features of each subgroup; (E) Reproducibility validation included subsampling stability analysis and stability testing across alternative brain parcellation templates.

This study proposes that EDs originate from dysregulation of core neurofunctional processes, with distinct subtypes exhibiting unique resting‐state FC patterns. To test this hypothesis, we employ NMF to decompose high‐dimensional FC data into interpretable disease factors through the following steps (Figure 1): (1) differential FC matrices between ED patients and healthy controls (HCs) are identified as input features. (2) NMF‐based dimensionality reduction is applied to these features to determine latent disease factors, quantify their FC signatures, and assign patients to subtypes based on individual factor loadings. (3) the clinical and cognitive relevance of each factor is analyzed by correlating representative functional networks (RFNs) with symptom profiles and functional decoding. (4) subtype‐specific characteristics—including demographic distributions, symptom severity, network‐level connectivity alterations and structural changes—are systematically compared. (5) the reproducibility of findings is examined through subsampling method and multi‐parcellation validation.

## 2 Materials and Methods

### 2.1 Subjects

This study initially included 480 participants (272 patients with EDs and 208 HCs). The AD subgroup comprised patients diagnosed with generalized anxiety disorder (GAD), panic disorder (PD), social anxiety disorder (SAD), and acrophobia (AC). After quality control, 18 participants exhibiting excessive head motion (translation > 3 mm or rotation > 3°) were excluded. Consequently, the final analytical sample consisted of 462 participants, including 261 ED patients and 201 HC (Tables S1 and S2).

### 2.2 Ethics Statement

All study procedures were conducted in accordance with the Declaration of Helsinki (1975). Written informed consent was obtained from all participants prior to study enrollment. Details regarding the dataset information are provided in the Method S1.

The data utilized in this study were sourced from three distinct datasets, each originating from separate research projects. The study involving AD was approved by the Ethics Committee of Nanjing Brain Hospital (Approval ID: 2019‐KY042‐01). The research on MDD received approval from the Ethics Committee of Nanjing Brain Hospital (Approval ID: 2019‐KY041‐01). The study concerning PTSD was approved by the Ethics Committees of the Second Xiangya Hospital of Central South University and Hainan General Hospital (Approval ID: 2013GJJ‐055).

### 2.3 Image Acquisition, Preprocessing and Feature Extraction

Resting‐state fMRI (rs‐fMRI) and T1 images were acquired across four distinct MRI scanners. Rs‐fMRI data were preprocessed using the DPABI toolbox (version 7.0, http://rfmri.org/dpabi) [18] and T1 data were preprocessed by Freesurfer (version 6.0, http://surfer.nmr.mgh.harvard.edu/). The corresponding MRI acquisition protocols and detailed preprocessing procedures are described in Methods S2, S3 and Tables S3, S4.

Following preprocessing, rs‐fMRI data were parcellated using the Power‐264 functional atlas [19]. Eight cerebellar regions were excluded to focus the analysis on cortical and subcortical networks, resulting in 256 non‐cerebellar regions of interest (ROIs). Regional mean time‐series were extracted, and pairwise Pearson correlation coefficients were computed to construct FC matrices, yielding 32,640 FC features per participant (256 × 255/2 = 32,640). Given the multisite nature of the data, ComBat harmonization (https://github.com/rpomponio/neuroHarmonize) was employed prior to feature selection to mitigate technical variance. Since dataset (site) and diagnostic groups are perfectly confounded in our cohort, ComBat was used strictly for variance stabilization (scale harmonization) rather than mean correction, ensuring that inter‐site heteroscedasticity did not bias subsequent statistical tests [20, 21].

FC differences between 261 patients and 201 HC were analyzed using two‐sample *t*‐tests (uncorrected *p* < 0.01). This uncorrected *p* < 0.01 threshold was chosen over more stringent corrections to prevent an excessive reduction in feature dimensionality, thereby ensuring that a sufficient number of biologically plausible connections was retained to support stable NMF decomposition. A total of 1098 significantly different FCs were identified, which were subsequently used as input features for clustering analysis.

### 2.4 NMF Clustering

To investigate ED subtypes, NMF [22] was applied to cluster the altered FC patterns of ED patients. This decomposition generated two matrices: (1) a basis matrix representing latent disease factors and (2) a coefficient matrix quantifying individual loading weights on these factors. Detailed NMF implementation procedures are provided in the Method S4A. For each latent factor, the top 5% of FC with the highest basis weights were defined as RFNs. To ensure robust subtype classification, the NMF algorithm was iterated 100 times. Final cluster labels were determined through iterative consensus, where each participant was categorized into the RFN demonstrating the highest mean coefficient weight across 100 NMF iterations (Figure 1B).

The optimal number of latent factors was determined through the evaluation of the cophenetic correlation coefficient (cluster stability), the silhouette score (separation quality), and the residual sum of squares (Method S4B) [23]. The NMF clustering algorithm was implemented using the NMF package [24] within the R statistical environment (version 4.4.1).

### 2.5 Functional Decoding and Clinical Relevance for RFNs

To elucidate the biological interpretations of RFNs across subtypes, we performed functional decoding (Figure 1C). Cognitive processes or behavioral functions were mapped to the brain regions within RFNs using probabilistic activation maps from Neurosynth (https://neurosynth.org/) [25]. Terms with ambiguous meanings (e.g., “field” and “valid”) were excluded from Neurosynth's repository (containing > 4000 search terms). 206 biologically meaningful terms were selected referring to the terms listed in Cheng et al.'s study [26]. For each RFN, Neurosynth's probabilistic framework was employed to calculate both forward inference estimates (the probability of cognitive states given observed activation patterns) and reverse inference estimates (the probability of activation patterns given cognitive states). Functional interpretations were derived from the top 10 highest‐ranking behavioral domains identified based on forward inference probability values, due to the superior reliability of forward inference.

Furthermore, we examined the associations between RFN patterns and clinical symptom severity by computing Spearman's rank correlations for each participant between the five RFN weights and their clinical scores including the Hamilton Anxiety Rating Scale (HAMA), Hamilton Depression Rating Scale (HAMD), Generalized Anxiety Disorder‐7 (GAD‐7), Self‐Rating Anxiety Scale (SAS), and Patient Health Questionnaire—Panic Disorder Module (PHQ‐PD) (Figure 1C). A nonparametric permutation test (1000 random shuffles of symptom scores) was employed to assess whether brain‐behavior correlations exceeded chance levels, with *p* values calculated from the resulting null distribution.

### 2.6 Subtype Characteristic Analyses

We systematically characterized diagnostic composition, demographic profiles, clinical assessments, and RFN features across five identified subtypes (Figure 1D). Between‐subtype comparisons of demographic and clinical variables were conducted using one‐way ANOVA. To examine RFN alterations between patient subtypes and HCs, FC matrices were first z‐transformed at the individual level, followed by two‐sample *t*‐tests with false discovery rate (FDR) correction (pFDR < 0.05). The proportion of significantly altered RFNs was quantified for each subtype. At the macroscale network level, we computed both intra‐network and inter‐network connectivity strengths across seven canonical functional networks and subcortical structures. Network‐wise comparisons between each patient subtype and HC were performed using independent samples *t*‐tests with FDR correction (pFDR < 0.05). Structural features, including GMV and cortical thickness, were also compared between each subtype and HC as well as between each subgroup and other ED patients. Age, gender, and total intracranial volume were entered as covariates to mitigate potential confounding effects.

### 2.7 Robustness Validation

To evaluate the stability of our subtyping framework against potential sampling biases, we conducted 100 iterations of NMF clustering using randomly selected 90% patient subsets. We computed: (1) factor composition stability using Spearman's correlation between original and resampled RFNs, (2) classification consistency rate: proportion of patients maintaining identical subtype labels across iterations, and (3) mean feature weights across all bootstrap iterations to obtain reidentified RFNs based on consensus weights (top 5% threshold preservation). Furthermore, for parcellation scheme validation, the entire analytical pipeline was replicated using the Dosenbach's 160‐node atlas [27], following the same clustering process as used for the aforementioned power‐264 template.

## 3 Results

### 3.1 Five Subtypes Identified in Emotional Disorders

**FIGURE 2:**
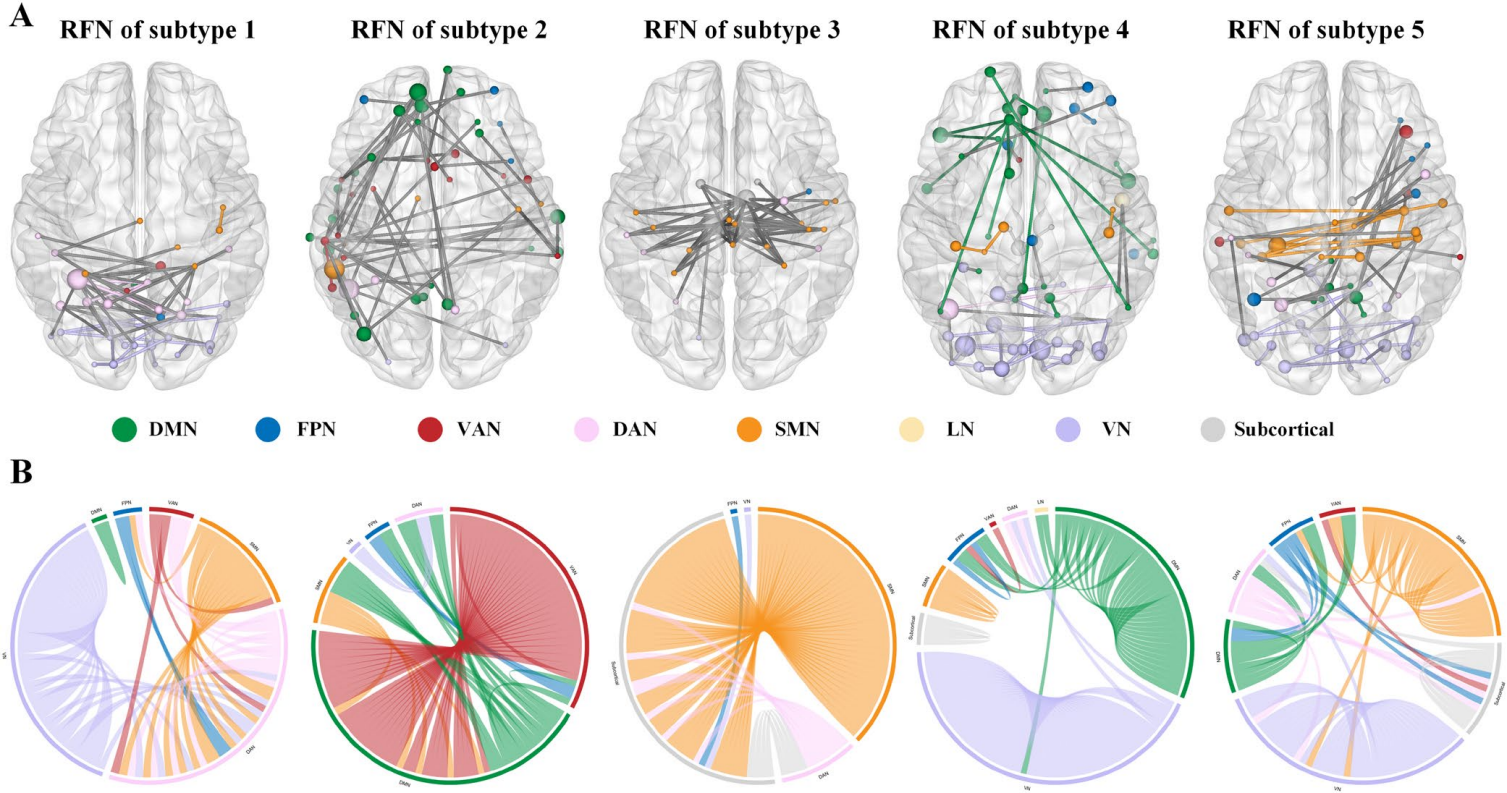
Identified 5 RFNs of ED patients. (A) Functional connectivity (FC) patterns of the 5 RFNs, displaying the top 5% of FC weights. Intra‐network connections are color‐coded by their respective intrinsic network, while inter‐network connections are depicted in gray; (B) Schematic representations of resting‐state brain networks corresponding to the 5 RFNs. DAN, dorsal attention network; DMN, default mode network; FPN, frontal parietal network; LN, limbic network; SMN, sensorimotor network; VAN, ventral attention network; VN, visual network.

NMF delineated five neurobiologically defined subtypes (ST1–ST5) among patients with ED, each characterized by unique FC patterns derived from the basis matrix weights (Results S1 and Figure S1). RFNs for each subtype comprised the top 5% of connections ranked by their basis weights (Figure 2).

Subtype 1 (ST1) was primarily defined by sensorimotor network (SMN)‐dorsal attention network (DAN) connectivity, including links between bilateral postcentral gyri and the left inferior parietal gyrus (IPG)/right precuneus, as well as DAN‐ventral attention network (VAN) integration involving connections between the IPG and the right parcentral lobule, alongside intra‐connections within visual network (VN) and DAN. Subtype 2 (ST2) featured VAN‐default mode network (DMN) and SMN‐DMN interactions, with prominent intra‐temporal connections spanning the superior/middle temporal gyri and insula, and integration of the left superior temporal gyrus with bilateral prefrontal regions. Subtype 3 (ST3) exhibited SMN‐subcortical connectivity focused on bilateral thalamic nuclei, specifically connections between the bilateral thalamus and precentral/postcentral gyrus, as well as thalamus‐supplementary motor area linkages. Subtype 4 (ST4) demonstrated intra‐network coherence, marked by the connections within classic DMN regions including prefrontal lobe, angular gyrus, anterior cingulate gyrus (ACC), and precuneus, as well as hierarchical visual system integration from primary to associative regions in the VN. Subtype 5 (ST5) displayed multi‐network‐subcortical integration, characterized by right insula‐rolandic operculum connections, links between thalamus and frontal, temporal, and parietal lobe, as well as frontoparietal network (FPN)‐DMN interactions involving IPG, precuneus, post cingulate cortex (PCC), and orbitofrontal cortex.

### 3.2 Behavioral and Symptomatic Correlates of the Five RFNs

Functional annotation of the five disease factors identified 206 biologically relevant cognitive/behavioral terms. RFN1 of factor 1 was primarily associated with visual motion processing and action observation, RFN2 with auditory processing and theory of mind, RFN3 with motor task and somatosensory, RFN4 with autobiographical memory retrieval, and Factor 5 with multisensory integration and visuospatial processing (detailed term distributions in Figure S2).

**FIGURE 3:**
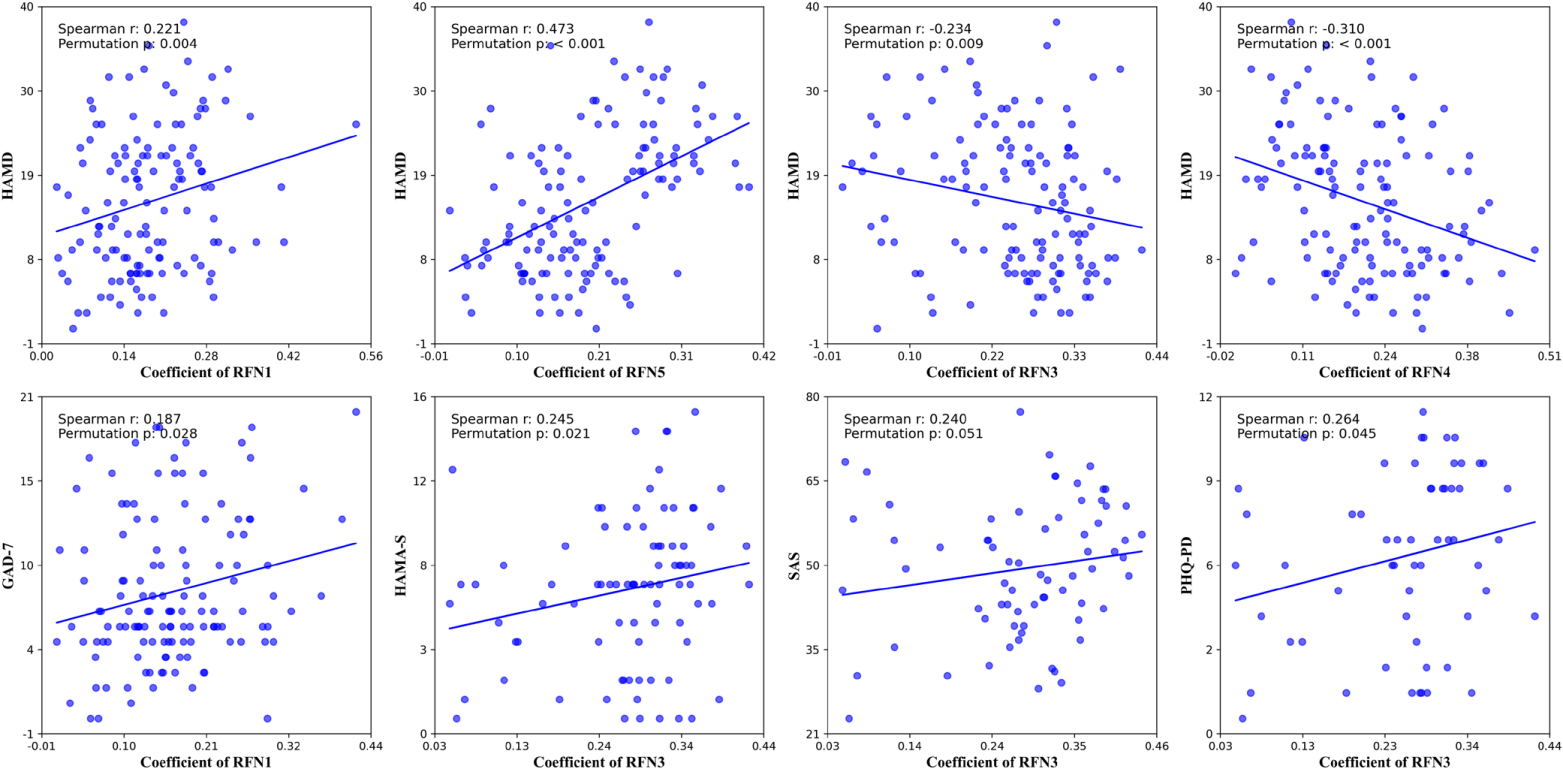
Spearman's correlation between RFN coefficient weights and clinical scale scores in ED patients. The coefficients are derived from the H matrix, generated by NMF of the connectivity matrix, and represent each participant's loading on every RFN. GAD‐7, Generalized Anxiety Disorder 7‐item Scale; HAMA‐S, Hamilton Anxiety Rating Scale—Somatic Subscale; HAMD, Hamilton Depression Rating Scale; PHQ‐PD, Patient Health Questionnaire—Panic Disorder Module; SAS, Self‐Rating Anxiety Scale.

Clinically, RFN weights exhibited differential correlations with symptom severity (Figure 3). Depressive symptoms, as measured by the HAMD, showed significant positive correlations with RFN1 (*r* = 0.221, *p* = 0.004) and RFN5 (*r* = 0.473, *p* < 0.001), while negative correlations were observed for RFN3 (*r* = −0.234, *p* = 0.009) and RFN4 (*r* = −0.310, *p* < 0.001). Anxiety symptoms displayed divergent associations: RFN1 was positively correlated with GAD‐7 scores (*r* = 0.187, *p* = 0.028). RFN3 demonstrated positive associations with somatosensory symptoms, including positive correlations with the somatic subscale of the Hamilton Anxiety Scale (HAMA‐S: *r* = 0.245, *p* = 0.021) and the PHQ‐PD (*r* = 0.264, *p* = 0.045). The correlation between RFN3 and SAS scores was not statistically significant after permutation testing (*r* = 0.240, *p* = 0.051).

### 3.3 Demographic and Symptomatic Characteristics Across Five Subtypes

**FIGURE 4:**
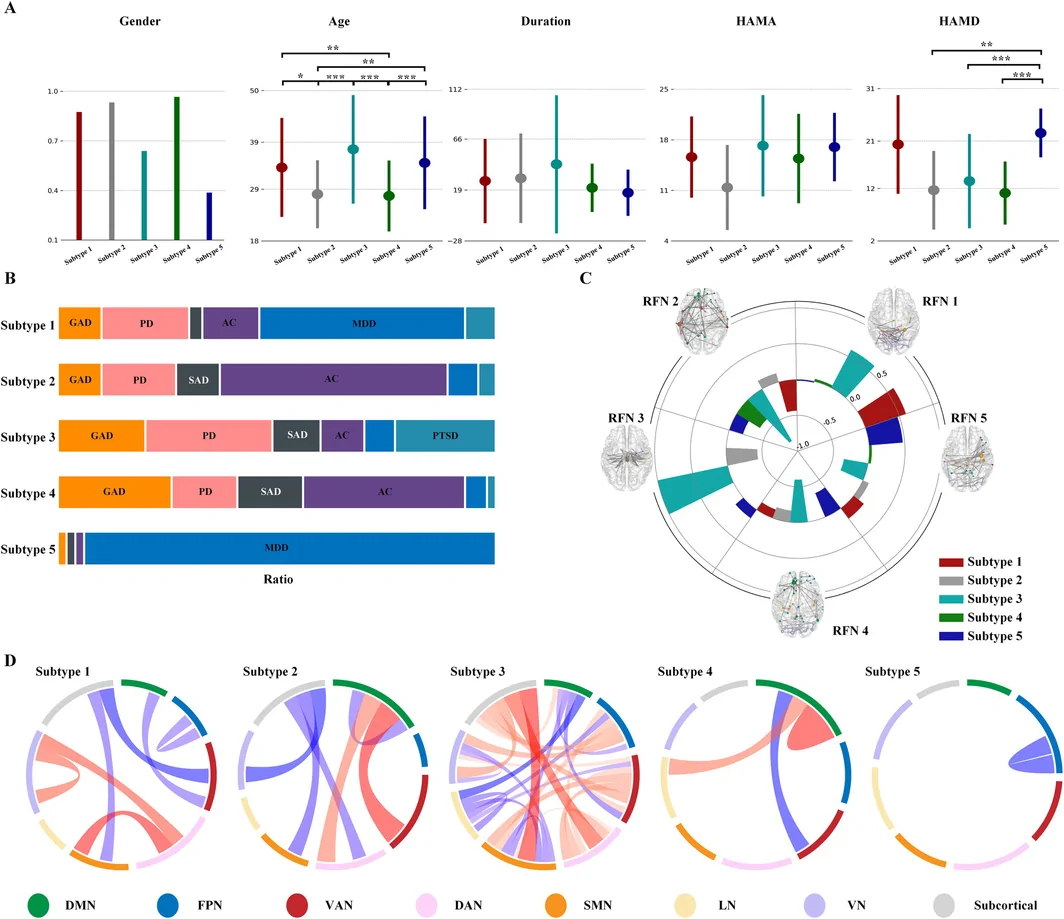
Demographic, symptomatic profiles, and altered RFN compositions and functional network connectivity across five subgroups compared to HC. (A) Demographic and clinical characteristics of the five subgroups (left to right): Gender distribution (male/female), age, disease duration, HAMA scores, and HAMD scores. Asterisks (*) indicate significant differences between the subgroup and HC (*p < 0.05; **p < 0.01; ***p < 0.001); (B) Diagnostic distributions within each subgroup; (C) Proportional changes in FC of the 5 RFNs relative to HC. Values > 0 indicate higher FC in the subgroup compared to HC; values < 0 indicate lower FC; (D) Altered network‐level connection strengths in subgroups versus HC (*pFDR* < 0.05). Red links denote stronger network connectivity in subgroups, while blue links denote weaker connectivity; color intensity reflects the magnitude of the *t*‐value. AC, acrophobia; DAN, dorsal attention network; FPN, frontal parietal network; GAD, generalized anxiety disorder; LN, limbic network; MDD, major depressive disorder; PD, panic disorder; PTSD, post‐traumatic stress disorder. DMN, default mode network; SAD, social anxiety disorder; SMN, sensorimotor network; VAN, ventral attention network; VN, visual network.

The clustering solution categorized 261 ED patients into five distinct neurophysiological subtypes: 30 patients (11.5%) were classified into ST1 (disease factor 1‐dominant), 29 (11.1%) into ST2, 100 (38.3%) into ST3, 59 (22.6%) into ST4, and 43 (16.5%) into ST5. Diagnostic composition across subtypes is illustrated in Figure 4B. ST1 comprised equal proportions of anxiety and depression diagnoses. ST2 and ST4 were predominantly composed of SAD and AC patients (> 50%). ST3 primarily included GAD, PD, and PTSD patients, whereas ST5 was dominated by MDD patients.

Demographic and symptomatic profiles are summarized in Figure 4A. Significant age differences emerged across subtypes (*F* = 11.454, *p* < 0.001), with ST2 patients being younger than ST1 (*p* = 0.030), ST3 (*p* < 0.001), and ST5 (*p* = 0.006). Similarly, ST4 patients were younger than ST1 (*p* = 0.008), ST3 (*p* < 0.001), and ST5 (*p* < 0.001). No significant gender (*χ* ^*2*^ = 5.835, *p* = 0.212) or illness duration (*F* = 1.414, *p* = 0.236) differences were observed. Clinically, while HAMA scores showed no group differences (*F* = 1.953, *p* = 0.106), significant variation emerged in HAMD scores (*F* = 9.926, *p* < 0.001), with ST5 exhibiting higher HAMD scores than ST2 (*p* = 0.009), ST3 (*p* < 0.001), and ST4 (*p* < 0.001).

### 3.4 RFN Alterations and Network Connectivity Profiles Across Five Subtypes

As shown in Figure 4C, Patient subgroups exhibited distinct FC patterns across all five RFNs compared to HCs. All subtypes except ST1 demonstrated reduced FC in RFN2 compared to HCs. ST1 displayed widespread FC elevation in RFN1, while ST2 showed marked FC reduction in RFN3. ST3 exhibited significant FC differences across all RFNs relative to HCs, characterized by increased FC in RFN1 and RFN3 but decreased FC in RFN2, RFN4, and RFN5. ST5 was distinguished by comprehensive FC elevation in RFN5 alongside significant FC reduction in RFN4. Spatial details of these FC changes are provided in Figure S3.

Network‐wise analyses revealed divergent connectivity patterns across subtypes (Figure 4D). ST1 patients showed increased connectivity between the DAN and SMN, but reduced connectivity between subcortical regions and VAN/SMN, as well as diminished DMN‐VAN connectivity and FPN intra‐connectivity. ST2 exhibited enhanced DMN‐VAN and DMN‐DAN integration alongside weakened intra‐DMN connectivity and reduced subcortical‐SMN/DAN links. ST3 demonstrated global hyperconnectivity between subcortical regions and all networks except the limbic network (LN), coupled with hypoconnectivity of DMN and LN with other networks. ST4 displayed DMN‐centric alterations, including strengthened intra‐DMN connectivity, DMN‐LN connectivity, and attenuated DMN‐VAN interactions. ST5 was characterized by a reduction in FPN intra‐connectivity.

### 3.5 Structural Attributes Across Five Subtypes

No significant differences in GMV were observed between the five subgroups and HC, nor among the five subgroups themselves. In contrast, cortical thickness analysis revealed that ST5 exhibited significantly greater thickness values compared to other subgroups in the following left‐hemisphere regions: the left fusiform gyrus, left inferior parietal lobe, left superior temporal lobe, left insula, and right supramarginal gyrus (Figure S4).

### 3.6 Validation Analyses

**FIGURE 5:**
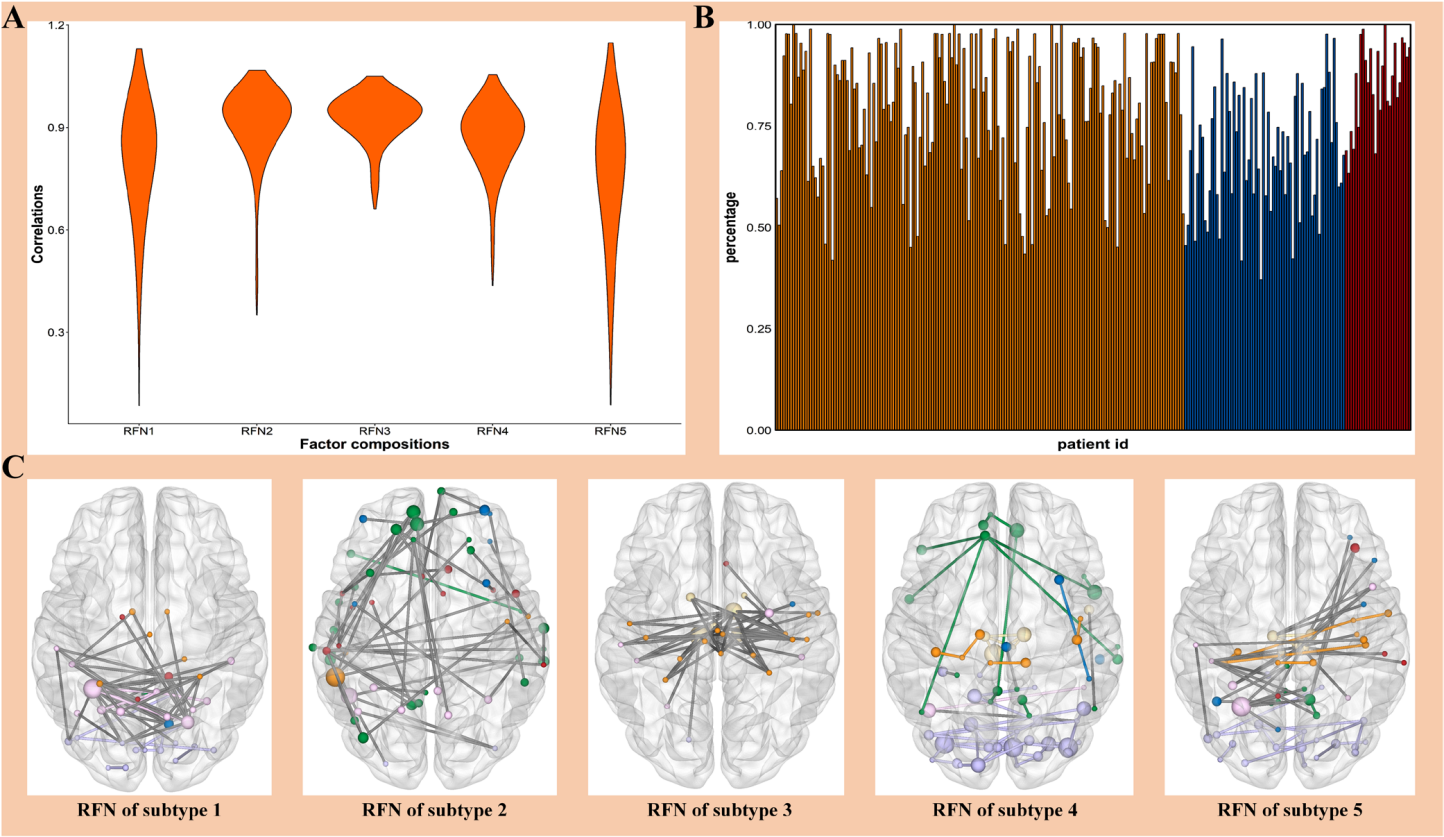
Reproducibility validation results. Consistency between the original results and 100 subsampling iterations (randomly selecting 90% of participants): (A) Distribution of correlations between RFN coefficient weights derived from subsampled datasets and the original weights; (B) Proportion of subsampling iterations with subgroup labels identical to the original clustering labels; (C) Spatial similarity between RFN compositions of the subsampling results and the original RFNs.

The reproducibility validation results are presented in Figure 5. High correlations were observed between the RFN factor compositions derived from the whole patient group and those obtained from NMF analyses using 90% subsets of patients. Mean correlation coefficients for RFN1, RFN2, RFN3, RFN4, and RFN5 were 0.806, 0.912, 0.930, 0.870, and 0.767, respectively (Figure 5A). Subtype assignment consistency across iterations was robust (Figure 5B), with high spatial overlap between the RFNs identified using the subsets and those derived from the full sample (Figure 5C). The results of parcellation scheme validation demonstrated that, among the five 5 RFNs, all except RFN5 exhibited cross‐parcellation consistency (Figure S5).

Furthermore, to assess potential confounding effects of demographic variables, we examined the relationship between RFN weights and both age and gender. We found no significant gender differences across any of the five RFN weights (all *p* > 0.05). For age, only RFN3 (*r* = 0.415, *p* < 0.001) and RFN4 (*r* = −0.341, *p* < 0.001) showed significant associations. We then performed partial correlation analyses between RFN weights and symptom scores, controlling for both age and gender. The majority of the significant brain‐behavior associations remained robust, supporting the validity of these pathological dimensions independent of age. The only exception was the correlation between RFN3 and SAS scores, which was marginal in the primary analysis (*p* = 0.051), became negligible and nonsignificant (*r* = 0.09, *p* = 0.465) after controlling for age and gender (Figure S6).

### 3.7 Sensitivity Analysis

To evaluate the empirical impact of the perfect cohort confound on feature selection, we performed a sensitivity analysis by omitting the ComBat step. Without variance stabilization, the number of statistically significant FC features expanded dramatically from 1098 to 3782, with a minimal overlap of only 137 features between the two sets. This substantial discrepancy indicates that omitting harmonization introduces massive false positives driven by site‐specific technical noise, whereas the ComBat‐stabilized features captured a more refined and biologically meaningful signal.

## 4 Discussion

To our knowledge, this study represents the first attempt to classify transdiagnostic subtypes of EDs based on resting‐state FC features. Our findings identified five distinct RFNs in ED patients, showing significant correlations with anxiety and depression severity. The five subtypes derived from these components exhibited marked differences in age, clinical symptoms, brain network patterns, and structural characteristics. These results provide neurobiological explanations for the comorbidity of anxiety and depression, offering empirical support for a dimensional classification system that transcends traditional diagnostic boundaries. The subtype‐specific network features may reflect individual neurofunctional states, thereby establishing a neuroimaging foundation for personalized treatment strategies in EDs.

Our study identified five shared disease factors across EDs, with ST3—associated with RFN3—encompassing the largest patient subgroup predominantly diagnosed with GAD, PD, and PTSD. RFN3, characterized by thalamus‐SMN connectivity, demonstrated significant positive correlations with panic symptoms, and the somatic anxiety subscale of the HAMA, alongside a negative correlation with HAMD scores (Figure 3). These findings suggest that somatosensory processing represents a core feature distinguishing AD and PTSD from MDD, consistent with prior reports of thalamic structural and functional impairments in ADs and PTSD [28, 29, 30, 31, 32]. The thalamus serves as a critical hub for relaying sensory, motor, and spatial signals to the cerebral cortex [33]. Connectivity between the thalamus and somatosensory cortex is implicated in interoceptive processing, where bodily signals are transmitted via the thalamus to the insula and subsequently integrated into higher‐order cognitive regions such as the prefrontal cortex [28, 34, 35, 36]. Aligning with neuroimaging evidence of interoceptive dysregulation in ADs [37, 38, 39, 40], ST3 patients exhibited the highest anxiety scores, longest illness duration, and the most complex network alterations. Although ST3 did not show statistically significant differences in HAMA scores or illness duration compared to other subtypes, these patients may represent a clinically distinct subgroup of ADs characterized by heightened interoceptive sensitivity and prominent somatic symptoms. This functional connectivity profile may be anchored in white matter microstructural and free‐water alterations, whose relationships with clinical symptoms or somatic traits have been shown to be significantly moderated by peripheral inflammatory status [41, 42]. Such a moderated biological pathway offers a plausible multilevel explanation for the somatic anxiety observed in ST3.

Notably, ST2 and ST4 patients displayed significantly reduced RFN3 connectivity compared to HC, indicating that not all AD patients exhibit enhanced interoceptive processing. This observation reconciles conflicting reports on interoceptive abnormalities in PD [39, 43], where heterogeneous subgroups may have been obscured by low statistical power in conventional case–control analyses. Our transdiagnostic, data‐driven clustering approach, combined with a relatively large sample size, successfully unmasked these subtype‐specific characteristics, highlighting the utility of neurobiologically informed classification frameworks.

Among the five identified RFNs, four demonstrated significant correlations with depression severity. RFN1 and RFN5 were positively associated with depression scores, whereas RFN3 and RFN4 showed negative correlations. RFN1, characterized by extensive connectivity between the SMN and DAN, corresponded to ST1, which comprised equal proportions of MDD and AD patients. In contrast, RFN5‐associated ST5 was predominantly composed of MDD patients. Crucially, our results demonstrate a “fractionation” rather than a “recapitulation” of clinical categories; the MDD population was split across multiple subtypes (61% in ST5, 21% in ST1, and 11% in ST3, with the remainder distributed elsewhere). To verify whether ST5 merely recapitulates the MDD diagnosis, we examined the RFN5‐depression correlation specifically within the MDD group. While RFN5 weight was significantly correlated with depressive symptoms across the entire cohort (*r* = 0.473, *p* < 0.001), this association vanished when analyzed strictly within the MDD group (*r* = −0.182, *p* = 0.243). This indicates that traditional diagnostic boundaries aggregate biological heterogeneity that obscures specific brain‐behavior links.

Despite this diagnostic overlap, the distinction between ST1 (mixed) and ST5 (pure‐MDD) phenotypes suggests that heightened attention to somatic sensations may represent a core trait distinguishing comorbid AD‐MDD cases from typical MDD. While prior studies have identified shared neural markers of anxiety and depression, such as structural and functional abnormalities in frontal‐limbic regions [12, 13], these alterations likely reflect general pathophysiological mechanisms common to EDs and thus were not captured in our RFN patterns. The ST1, with mixed MDD and AD diagnoses, may represent an MDD subgroup marked by prominent anxiety symptoms, exhibiting enhanced connectivity in somatosensory‐related regions [44, 45]. Distinct from the mixed diagnoses in ST1, ST5 was characterized by a pure‐MDD population with significantly higher depression scores. Structurally, ST5 exhibited significantly greater cortical thickness in regions like the left insula and inferior parietal cortex compared to other subgroups, while showing no deviations from HCs. This preference for thickness over gray matter volume aligns with evidence that neuroanatomical heterogeneity in depression is more sensitively captured by thickness metrics, particularly within insular regions [46]. Furthermore, this localized thickening may reflect a specific biotype characterized by extreme positive deviations; large‐scale normative modeling has demonstrated that such structural anomalies are robustly linked to greater depressive symptom severity [47].

Of particular note, RFN4 was characterized by enhanced intra‐network connectivity, particularly within the self‐referential processing‐related DMN. All subtypes except ST4 exhibited reduced RFN4 FC compared to HC, indicating that impairments in self‐referential processing circuits are not specific to a single emotional disorder (e.g., MDD) but are broadly present across EDs. Reduced DMN connectivity involving the medial prefrontal cortex mPFC, ACC, angular gyrus, and precuneus has been widely reported in MDD patients [48, 49, 50, 51]. The DMN has been extensively studied in MDD due to its critical role in rumination symptoms [52, 53]. However, importantly, DMN abnormalities are not confined to MDD. In ADs, alterations in DMN connectivity have also been associated with sleep disturbances and anxiety severity [54, 55, 56]. This dual evidence underscores the transdiagnostic relevance of self‐referential processing across emotional disorders (EDs) and highlights the necessity of cross‐diagnostic research paradigms.

In contrast, ST4—associated with RFN4—displayed increased intra‐DMN connectivity, strengthened DMN‐limbic network (LN) integration, and attenuated DMN‐VAN interactions. Clinically, ST4 patients exhibited higher anxiety and lower depression severity compared to other subtypes. This suggests that hyperactive self‐referential processing may drive anxiety symptoms, specifically anticipatory worry and hypervigilance, rather than the depressive rumination typically associated with DMN hypoconnectivity, presenting a neurofunctional profile distinct from the DMN hypoconnectivity observed in other subtypes. These divergent DMN alterations highlight the spectrum of self‐processing abnormalities in EDs, where both excessive and diminished DMN engagement can manifest as clinically distinct phenotypes.

Intriguingly, RFN2—identified as one of the disease factors—showed no significant correlations with anxiety or depression severity. However, it exhibited widespread FC reductions across all subtypes except ST2, which was characterized by the mildest symptom profiles and predominantly comprised SAD and AC patients. Network‐wise, ST2 demonstrated enhanced connectivity between the DMN and attention networks alongside reduced FC between subcortical structures and other networks, suggesting a neurofunctional state of heightened environmental vigilance. RFN2 is linked to theory of mind (ToM), classically defined as the ability to represent mental states of oneself and others [57]. The observed FC reductions in RFN2 among patients with more severe anxiety and depression imply that this network's relative functional preservation might be linked to adaptive socio‐cognitive dimensions, such as theory of mind (ToM). While our Neurosynth decoding points toward a broad functional annotation involving ToM, we caution against a direct behavioral interpretation in the absence of direct socio‐cognitive or longitudinal data. It is worth noting that ToM impairment is well‐documented across psychiatric conditions [58, 59, 60, 61, 62, 63]. ED patients with stronger ToM‐related network integrity exhibited lower anxiety and depression scores, underscoring its potential role in resilience. This pattern suggests a potential link between the relative functional integrity of this system and clinical phenotypic variations, which warrants further investigation with dedicated behavioral batteries to confirm its hypothesized role in resilience.

The application of NMF for latent factor analysis and subtyping has been extensively utilized in psychiatric disorders such as autism spectrum disorder [17, 64] and MDD [65, 66], providing a dimensional framework grounded in objective neuroimaging biomarkers. In this study, we employed NMF to perform transdiagnostic subtyping of EDs, identifying five disease factors that dissect symptom manifestations at the network level. These factors reveal both transdiagnostic pathological mechanisms (e.g., thalamocortical hyperactivity of ST3) and protective neural signatures (e.g., preserved ToM‐related networks of ST2).

Methodologically, the NMF‐derived subtypes demonstrated robustness against demographic confounds. While age was significantly correlated with RFN3 and RFN4, the persistence of significant brain‐depression correlations after controlling for age supports the validity of these subtypes as capturing core pathological dimensions extending beyond demographic variation. A major methodological constraint of this study is the perfect collinearity between scanning sites and diagnostic labels. However, several lines of downstream evidence robustly validate the biological authenticity of our findings, arguing against the presence of mathematical artifacts. First, if our pipeline failed to resolve scanner‐specific variances, the downstream NMF would rigidly segregate cohorts according to scanning sites. Instead, we observed a subtype comprising a roughly balanced mixture of MDD and AD patients derived from different scanning sites, indicating that the decomposition reflects shared biological dimensions that transcend site boundaries. Second, because ComBat and NMF were performed without access to clinical ratings, the fact that 4 out of 5 RFN weights showed significant continuous linear correlations with depression severity across the cohort provides strong empirical support that the retained dimensions represent transdiagnostic axes of disease burden rather than redundant recapitulations of diagnostic categories or scanner effects.

However, despite these insights, our findings should be interpreted with caution due to several limitations. First, while using patient‐control differential FCs as NMF inputs effectively focuses the decomposition on disease‐related variance—as established in prior subtyping research [67]—we acknowledge that this supervised feature selection step may theoretically enrich for general patient‐control differences. Although our 90% subsampling validation demonstrated high internal stability, future studies could further validate these subtypes using cross‐validated feature selection approaches in larger, independent cohorts. Second, the imbalanced distribution of diagnostic groups in our sample—with ADs outnumbering MDD and PTSD cases—may have obscured subtype‐specific features of underrepresented groups during model construction. Third, the reliance on anxiety and depression symptom scales alone limits the phenotypic granularity critical for transdiagnostic research; future studies could enhance validity by incorporating psychometric tools tailored to the identified disease factors. Finally, the transdiagnostic design of this study poses challenges for external validation and reproducibility, given the scarcity of existing datasets encompassing multiple diagnostic categories. Although permutation tests confirmed that our brain‐behavior correlations were robust against random noise, future studies with larger, independent transdiagnostic cohorts remain essential to fully establish the external generalizability of these subtyping boundaries.

## 5 Conclusions

Overall, transdiagnostic research offers significant promise for elucidating the fundamental nature of psychiatric disorders. Our findings identified five transdiagnostic RFNs across EDs, delineating five distinct neurobiological subtypes. These results provide novel insights into the neuroimaging mechanisms underlying EDs from the perspective of objective biological disease factors and provide an explanatory framework linking brain network architecture to behavioral symptomatology and clinical diagnosis.

## Author Contributions

**Yun Wu:** conceptualization, methodology, formal analysis, validation, writing – original draft, writing – review and editing. **Chun Wang:** conceptualization, data curation, investigation, writing – review and editing. **Changjun Teng:** investigation, resources, validation. **Yan Lu:** software, formal analysis, validation, visualization; Dr. Lu also optimized the core algorithm codes and implemented advanced statistical validation procedures to thoroughly address the reviewers' critical concerns regarding the model's stability and robustness. **Chengbin Guan:** conceptualization, investigation, data curation, supervision, writing – review and editing. **Yuan Zhong:** conceptualization, methodology, supervision, funding acquisition, project administration, writing – review and editing.

## Funding

This research was funded by the National Natural Science Foundation of China (No. 32571269, 82371556), National Key R&D Program of China (Grant No. 2023YFC3341300), Natural Science Foundation of Jiangsu Province (No. BK20241881), “333 Talent Project” of Jiangsu Province, Health Science and Technology Development of Nanjing (YKK23145) and Postgraduate Research & Practice Innovation Program of Jiangsu Province (No. KYCX24_1720).

## Conflicts of Interest

The authors declare no conflicts of interest.

## Supporting information


**Table S1:** Demographic information of the participants.
**Table S2:** Number of participants included in the subgroup comparisons of clinical assessments.
**Table S3:** MRI scanner and image‐acquisition protocol information for the resting‐state datasets.
**Table S4:** MRI scanner and image‐acquisition protocol information for the T1 datasets.
**Figure S1:** The cophenetic coefficient, silhouette coefficient and RSS change in ED patients.
**Figure S2:** Functional decoding results of five identified RFNs.
**Figure S3:** Specific FC alterations between subtypes and HC.
**Figure S4:** Comparison of cortical thickness between ST5 and other subtypes.
**Figure S5:** Validation of RFNs based on Dosenbach's 160 parcellation scheme.
**Figure S6:** Partial correlation between RFN coefficient weights and clinical scale scores in ED patients, controlling for age and gender.

## Data Availability

The datasets used in this study are available from the corresponding author upon request.
