# Supplementary material for: Decoding Transdiagnostic Subtypes in Emotional Disorders
Source: CNS Neurosci Ther. 2026 Sep 1;32(9):e71122. doi: 10.1002/cns.71122 (PMC13531014; doi:10.1002/cns.71122)
Supplement: Supplementary file 1 [file CNS-32-e71122-s001.docx]

**Supplemental Information**

**Supplementary Methods**

**Method S1. Participants**

**AD dataset**

**MDD dataset**

**PTSD dataset**

**HC dataset**

**Method S2. Image acquisition**

**Method S3. Image preprocessing**

**Method S4. Non-negative Matrix Factorization (NMF) clustering**

**Method S4A. NMF Implementation**

**Method S4B. Determination of Optimal Cluster Number**

**Supplementary Results**

**Results S1. Determination of Optimal Cluster Number**

**Supplementary Tables**

**Table S1. Demographic information of the participants**

**Table S2. Number of Participants Included in the subgroup comparisons of clinical assessments**

**Table S3. MRI scanner and image-acquisition protocol information for the resting-state datasets**

**Table S4. MRI scanner and image-acquisition protocol information for the T1 datasets**

**Supplementary Figures**

**Figure S1. The cophenetic coefficient, silhouette coefficient and RSS change in ED patients.**

**Figure S2. Functional decoding results of five identified RFNs**

**Figure S3. Specific FC alterations between subtypes and HC**

**Figure S4. Comparison of cortical thickness between ST5 and other subtypes**

**Figure S5. Validation of RFNs based on Dosenbach's 160 parcellation scheme**

**Figure S6. Partial correlation between RFN coefficient weights and clinical scale scores in ED patients, controlling for age and gender**

**Supplementary References**

**Supplementary Methods**

**Method S1. Participants**

A total of 480 participants were included in this study, comprising 272 individuals with emotional disorders (ED) and 208 healthy controls (HC). Specifically, there were 178 anxiety disorder (AD) patients, 67 major depressive disorder (MDD) patients, and 27 post-traumatic stress disorder (PTSD) patients. Among the anxiety patients, 48 had generalized anxiety disorder (GAD), 50 had panic disorder (PD), 25 had social anxiety disorder (SAD), and 55 had specific phobias (acrophobia). Patients with AD and MDD were primarily recruited from Nanjing Brain Hospital, whereas participants with PTSD were derived from typhoon-affected communities in Wenchang City, Hainan Province. The HC group primarily consisted of normal individuals without mental illnesses who were recruited and matched at various sites for inclusion in the study. Considering the impact of head movement, 18 participants (6 GAD patients, 1 PD patient, 3 acrophobia patients, 1 MDD patient, and 7 HCs) were excluded from subsequent analyses, resulting in a final sample of 462 participants (261 with ED and 201 HC). Basic information of the participants is presented in Table S1. Detailed information, including ethics and the inclusion criteria, can be found elsewhere in our previous studies and the following description for specific datasets. Chi-square test results indicated significant age differences among the groups (*p* < 0.001), but no significant gender differences (*p* = 0.057).

In this study, the Hamilton Depression Rating Scale (HAMD) and Hamilton Anxiety Rating Scale (HAMA) were used to measure clinical symptoms of the patients. Due to the broad data sources and limitations of the initial project experimental design, not all patients were included in the subsequent scale-related analyses. Details of the participants included in the analysis are shown in Table S2.

**AD dataset**

The AD dataset was derived from a previous research project on the neural mechanisms and psychotherapy of AD patients at the Nanjing Brain Hospital of Nanjing Medical University. The current study utilized the baseline data within this dataset. The study was approved by the Ethics Committee of the Nanjing Brain Hospital (Ethics Approval ID: 2019-KY042-01). All participants signed a paper informed consent form after being fully informed. All participants with different diagnoses of anxiety were recruited based on the DSM-Ⅳ through the clinics of Nanjing Brain Hospital.

The exclusion criteria were as follows: (1) neurological disease, (2) more than one target diagnosis, including psychiatric and personality disorders, (3) severe physical illness, pregnancy, and/or breastfeeding, (4) use of psychiatric drugs or psychological counseling within six months, (5) suicidal risk, (6) MRI contraindications, and (7) major life change in the last year as defined by death of spouse, unemployment, severe illness, serious injury, legal disputes, property loss, traffic accident, natural disasters, or divorce.

A total of 101 HCs matched with the patient group in the AD dataset were recruited through outpatient advertisements and included in the HC dataset.

Related publications can be found elsewhere (Ding et al., 2022; Ma et al., 2019; Ma et al., 2020; Pang et al., 2021; H. Wu et al., 2022; Y. Wu et al., 2022; H. Xu et al., 2021; L. Xu, Xu, Ding, Li, & Wang, 2021; Yu et al., 2022).

**MDD dataset**

The MDD dataset originated from a previous intervention study on psychotherapy and pharmacotherapy for first-episode MDD patients at the Nanjing Brain Hospital affiliated with Nanjing Medical University. This study employed baseline data from all patients. The research was approved by the Ethics Committee of Nanjing Brain Hospital (Ethics Approval ID: 2019-KY041-01). All participants provided written informed consent after being fully informed. First-episode MDD patients were diagnosed by experienced psychiatrists according to the DSM-IV guidelines and confirmed through the Structured Clinical Interview for DSM-IV (SCID).

Inclusion criteria for the patient group included: (1) meeting DSM-IV diagnostic criteria for MDD; (2) aged 20-50 years; (3) first episode; (4) a score of ≥18 on the 24-item HAMD scale; (5) right-handed; (6) voluntary participation and signed informed consent. Exclusion criteria were as follows: (1) neurological diseases or other mental illnesses; (2) severe physical diseases or infectious disease; (3) serious suicide attempt or suicidal behavior; (4) drug or alcohol dependence; (5) bipolar disorder; (6) personality disorders; (7) MRI contraindications; (8) breastfeeding or pregnancy. Termination criteria: (1) no psychological treatment for more than 4 weeks; (2) suicide, refusal of food, stupor or psychotic symptoms occurred; (3) unwillingness to continue treatment.

A total of 27 HCs matched with the patient group in the MDD dataset were recruited through outpatient advertisements and included in the HC dataset for the current experiment.

Related publications can be found elsewhere (Hao et al., 2020; Li et al., 2019; Teng, Liu, Zhang, Zhong, & Wang, 2022; Z. Wu et al., 2022; Wu et al., 2020; Zhou et al., 2021).

**PTSD dataset**

The PTSD dataset includes PTSD patients recruited from November 2014 to January 2015. In 2014, a tropical cyclone named Rammasun, classified as a Category 4 storm, struck Wenchang City in Hainan Province, southern China. The participants were individuals from the affected surrounding areas who, after screening with the PTSD Checklist-Civilian Version (PCL), met the diagnostic criteria for PTSD according to DSM-IV. Approval was provided by the Ethics Committee of the Second Xiangya Hospital of Central South University and Hainan General Hospital (Ethics Approval ID: 2013GJJ-055). Signed consent was collected from all the PTSD patients after they were fully informed of the study description.

The following exclusion criteria were applied: (1) age under eighteen or above sixty-five; (2) significant neurological and medical conditions; (3) history of head injury or loss of consciousness; (4) left-handedness; (5) current comorbid or lifetime comorbid psychiatric disorders other than depression and anxiety; (6) psychotropic medication use; (7) alcohol or substance abuse; (8) contraindications for MRI, such as pregnancy, claustrophobia, or ferromagnetic implants.

A total of 32 HCs matched with the patient group in the PTSD dataset were recruited from the surrounding areas affected by the typhoon and included in the HC dataset for the current experiment.

Related publications can be found elsewhere (F. Chen et al., 2018; H. J. Chen et al., 2021; H. J. Chen et al., 2019; Dai et al., 2023; Ke et al., 2018; Liu et al., 2021; Weng et al., 2018; Xiao et al., 2023; T. Xu et al., 2022).

**HC dataset**

The HC dataset consists of HC participants recruited from the aforementioned studies as well as another dataset (HC-1) of 46 HCs. This dataset originated from a previous study on the genes, imaging mechanisms, and therapeutic effects in patients with obsessive-compulsive disorder at the Nanjing Brain Hospital affiliated with Nanjing Medical University. The research was approved by the Ethics Committee of Nanjing Brain Hospital (Ethics Approval ID: 2023-KY073-01). Related publications can be found elsewhere (Zhang et al., 2024).

The exclusion criteria included MRI related exclusion criteria, current psychiatric/neurological disorders, use of psychiatric drugs in the past three months prior to scanning, pregnancy and a history of head trauma.

**Method S2. Image acquisition**

The individuals were scanned on 3T scanners using standardized protocols at each site. Individuals were scanned on one of four different MRI scanners to obtain resting-state functional images and 3D T1-weighted images. The corresponding MRI acquisition protocols are described in Table S3 & S4. To minimize the variation in head motion, we excluded the subjects with large head motion in any direction corresponding to translation >3 mm or any rotation >3°.

**Method S3. Image preprocessing**

The preprocessing of rs-fMRI data was performed using the DPABI V7.0 software package (<http://rfmri.org/dpabi>) based on the Matlab platform (<http://www.brain-connectivity-toolbox.net>) (Yan, Wang, Zuo, & Zang, 2016), and included the following steps: (1) the first 10 time points were discarded; (2) slice-timing correction; (3) realignment to the first volume; (4) covariate regression (including Friston’s twenty-four head motion parameters and confounding signals from white matter and cerebrospinal fluid); (5) normalization using each participant’s T1 image unified segmentation; (6) smoothing with a Gaussian kernel (6 mm FWHM); (7) temporal band-pass filtering with a cut-off frequency of 0.01-0.1 Hz.

The preprocessing of T1-weighted data was carried out using Freesurfer (version 6.0, available at <http://surfer.nmr.mgh.harvard.edu/>). The recon-all processing pipeline encompasses motion correction, extraction of brain tissue, segmentation, and parcellation into regions of interest (Desikan et al., 2006). FreeSurfer's morphometric procedures have demonstrated robust test-retest reliability across different scanner manufacturers (Han et al., 2006). Structural features extracted for subgroup description included gray matter volume (GMV) and cortical thickness values for each region. Prior to comparison, Combat harmonization (<https://github.com/rpomponio/neuroHarmonize>) was performed to control for multi-site variability, with age, gender, and clinical diagnosis included as covariates (Fortin et al., 2018; Johnson, Li, & Rabinovic, 2006).

**Method S4. Non-negative Matrix Factorization (NMF) clustering**

**Method S4A. NMF Implementation**

NMF decomposes a non-negative matrix **X** into two factor matrices: the basis matrix **W** and the coefficient matrix **H**, following the relationship **X ≈ W × H** (Lee & Seung, 1999). This data-driven approach identifies latent biological patterns by clustering high-dimensional biological features into distinct profiles (represented by **W**) and quantifying individual participant loadings across these clusters (encoded in **H**). In this study, the input matrix **X** was structured as a 1,098 × 261 matrix, where the 1,098 rows correspond to the group-discriminative FCs and the 261 columns represent the ED patients. The basis matrix **W** (dimensions: 1,098 × k, where k is the number of clusters) captures the weight of each FC feature within each cluster, with the top 5% of features by weight in each cluster defined as its representative functional network (RFN). The coefficient matrix **H** (dimensions: k × 261) contains the loading weights of each patient across all clusters, enabling subtype classification based on the cluster with the highest weight for each individual.

To satisfy the non-negativity requirement of NMF, all Pearson’s correlation coefficients of FC were normalized to the range [0, 1] using min-max scaling across participants. This transformation ensured compatibility with the algorithm while preserving relative differences in connectivity strength. The non-smooth NMF (nsNMF) algorithm was selected to enhance the sparsity of the cluster solutions, thereby improving the interpretability of the derived RFNs (Pascual-Montano, Carazo, Kochi, Lehmann, & Pascual-Marqui, 2006). By promoting sparsity in the basis matrix, nsNMF facilitates the identification of distinct functional networks, which is critical for delineating clinically meaningful subtypes. This methodological choice aligns with the goal of extracting clear neurobiological signatures from high-dimensional FC data while maintaining robustness against noise.

**Method S4B. Determination of Optimal Cluster Number**

The optimal number of clusters was determined through a comprehensive evaluation of three metrics: cophenetic coefficient, silhouette coefficient, and residual sum of squares (RSS). To validate the robustness of these metrics, synthetic datasets mimicking the statistical structure of the original data were generated, and the NMF algorithm was applied iteratively (1,000 repetitions) to establish baseline distributions for comparative analysis.

The cophenetic coefficient quantifies the stability of clustering solutions, decreases progressively as the cluster number increases (Brunet, Tamayo, Golub, & Mesirov, 2004) . The silhouette coefficient measures intra-cluster cohesion versus inter-cluster separation (Lovmar, Ahlford, Jonsson, & Syvänen, 2005). The value ranges from -1 to 1. A higher silhouette coefficient indicates a greater level of consistency within each cluster and more pronounced differences between clusters. RSS represents the unexplained variance in the NMF model, decreases monotonically with increasing cluster numbers. By comparing the RSS changes between real data and randomly generated data, we can ascertain whether the heightened explanation of variance by diverse rank solutions is attributable to genuine clustering patterns or merely the result of noise decomposition (Frigyesi & Höglund, 2008).

**Supplementary Results**

**Results S1. Determination of Optimal Cluster Number**

The cophenetic coefficient declined sharply for cluster numbers below 4 but plateaued beyond 4 clusters, with values remaining above the empirical stability threshold (≥0.75). Notably, a slight rebound in stability was observed at cluster number 7 and 8, approximating the stability level of cluster number 5 (Figure S2). To balance stability with parsimony, cluster number 5 was prioritized.

The silhouette coefficient stabilized at cluster numbers ≥5 (Figure S2). Values plateaued near 0.4, reflecting moderate but consistent classification quality. While higher values are ideal, this stabilization indicates diminishing returns in cluster separation beyond 5, supporting 5 as a pragmatic choice to avoid overfitting while retaining meaningful biological distinctions.

The curve of RSS changes showed two significant inflection points: a marked reduction from 4 to 5 clusters and a smaller decline from 6 to 7 clusters. Beyond 7 clusters, RSS reductions aligned with noise levels observed in synthetic datasets. These inflection points suggested that cluster numbers 4, 5, and 6 captured critical biological variances.

Therefore, after considering three metrics comprehensively, we have chosen 5 as the optimal number of clusters.

**Supplementary Tables**

**Table S1. Demographic information of the participants**

| **Groups** | | **N** | **age** | ***p*** | **Gender (M/F)** | ***p*** |
| --- | --- | --- | --- | --- | --- | --- |
| **AD** | **GAD** | **42** | **34.52±9.53** |  | **24/18** | **0.057** |
|  | **PD** | **49** | **35.12±8.67** |  | **25/24** |  |
|  | **SAD** | **25** | **26.80±6.92** |  | **10/15** |  |
|  | **acrophobia** | **52** | **23.67±3.32** | **<0.001** | **15/37** |  |
| **MDD** | | **66** | **34.92±9.67** |  | **27/39** |  |
| **PTSD** | | **27** | **48.41±10.32** |  | **7/20** |  |
| **HC** | | **201** | **29.99±11.26** |  | **84/117** |  |

**Table S2. Number of Participants Included in the subgroup comparisons of clinical assessments**

| **Groups** | | **N** | **HAMD** | **HAMA** | **GAD-7** | **SAS** | **PHQ-PD** |
| --- | --- | --- | --- | --- | --- | --- | --- |
| **AD** | **GAD** | **42** | **30** | **33** | **25** | **14** | **10** |
|  | **PD** | **49** | **32** | **34** | **31** | **5** | **26** |
|  | **SAD** | **25** | **25** | **25** | **25** | **25** | **23** |
|  | **acrophobia** | **52** | **0** | **0** | **48** | **0** | **0** |
| **MDD** | | **66** | **43** | **33** | **0** | **0** | **0** |
| **PTSD** | | **27** | **0** | **0** | **0** | **27** | **0** |
| **all** | | **201** | **130** | **125** | **129** | **72** | **59** |

HAMD = Hamilton Depression Rating Scale; GAD-7 = Generalized Anxiety Disorder 7-item Scale; HAMA = Hamilton Anxiety Rating Scale; SAS = Self-Rating Anxiety Scale; PHQ-PD = Patient Health Questionnaire - Panic Disorder Module.

**Table S3. MRI scanner and image-acquisition protocol information for the resting-state datasets**

| Site | Field of  strength | Brand | Protocol  name | Repetition  time | Echo  time | Flip  angle | Field of view | Matrix | Slice number  /thickness (gap) |
| --- | --- | --- | --- | --- | --- | --- | --- | --- | --- |
| AD | 3.0 T | Siemens Verio | EPI | 2 s | 30 ms | 90 | 240 × 240 | 64 × 64 | 36/4 (0) |
| MDD | 3.0 T | Siemens Verio | EPI | 3 s | 40 ms | 90 | 240 × 240 | 64 × 64 | 32/4 (4) |
| PTSD | 3.0 T | Siemens Skyra | EPI | 2 s | 30 ms | 90 | 230 × 230 | 64 × 64 | 35/3.6 (0) |
| HC-1 | 3.0 T | Siemens Discovery | EPI | 2 s | 30 ms | 90 | 100 × 100 | 64 × 64 | 32/4 (4) |

**Table S4. MRI scanner and image-acquisition protocol information for the T1 datasets**

| Site | Field of  strength | Brand | Protocol  name | Repetition  time | Echo  time | Flip  angle | Field of view | Matrix | Slice number  /thickness (gap) |
| --- | --- | --- | --- | --- | --- | --- | --- | --- | --- |
| AD | 3.0 T | Siemens Verio | MP-RAGE | 1900 ms | 2.48 ms | 9 | 250 × 250 | 256 × 256 | 176/1 (0.5) |
| MDD | 3.0 T | Siemens Verio | MP-RAGE | 1900 ms | 2.48 ms | 9 | 250 × 250 | 256 × 256 | 176/1 (0.5) |
| PTSD | 3.0 T | Siemens Skyra | MP-RAGE | 2300 ms | 1.97 ms | 9 | 256 × 256 | 256 × 256 | 176/1 (0) |
| HC-1 | 3.0 T | Siemens Discovery | MP-RAGE | 8.2 ms | 3.2 ms | 12 | 256 × 256 | 256 × 256 | 172/1 (1) |

**Supplementary Figures**

**Figure S1. The cophenetic coefficient, silhouette coefficient and RSS change in ED patients.** "Real Data" of Datatype refers to the real data of functional connectivity and the "Random Data" refers to the random data that has the same shape as the true data.

**Figure S2. Functional decoding results of five identified RFNs.** A deeper color and a larger font size signify higher probability values.

**Figure S3. Specific FC alterations between subtypes and HC. (A)** The differences in FC of the 5 RFNs between each subgroup and the HC group (*pFDR* < 0.05). Red lines represent FCs in the subgroups that is stronger than that of the HC group, whereas blue lines represent FCs in the subgroups that is weaker than that of the HC group; **(B)** The network distribution corresponding to the FC differences between each subgroup and the HC group. Similarly, red lines indicate FCs in the subgroups that is stronger than that of the HC group, while blue lines indicate FCs in the subgroups that is weaker than that of the HC group. DMN = default mode network; FPN = frontal parietal network; VAN = ventral attention network; DAN = dorsal attention network; SMN = sensorimotor network; LN = limbic network; VN = visual network.

**Figure S4. Comparison of cortical thickness between ST5 and other subtypes.** The ST5 group exhibited significantly greater cortical thickness in several regions compared to the other subtypes: the left fusiform gyrus, the left inferior parietal lobe, the left superior temporal lobe, the left insula, and the right supramarginal gyrus (*pFDR* < 0.05).

**Figure S5. Validation of RFNs based on Dosenbach's 160 parcellation scheme. ‌(A)**‌ Functional connectivity (FC) patterns of the 5 RFNs, displaying the top 5% of FC weights. Intra-network connections are color-coded by their respective intrinsic network, while inter-network connections are depicted in gray; **‌(B)**‌ Schematic representations of resting-state brain networks corresponding to the 5 RFNs. DMN = default mode network; FPN = frontal parietal network; VAN = ventral attention network; DAN = dorsal attention network; SMN = sensorimotor network; LN = limbic network; VN = visual network.

**‌Figure S6. Partial correlation between RFN coefficient weights and clinical scale scores in ED patients, controlling for age and gender.**‌ The coefficients are derived from the H matrix, generated by NMF of the connectivity matrix, and represent each participant's loading on every RFN. HAMD = Hamilton Depression Rating Scale; GAD-7 = Generalized Anxiety Disorder 7-item Scale; HAMA-S = Hamilton Anxiety Rating Scale - Somatic Subscale; SAS = Self-Rating Anxiety Scale; PHQ-PD = Patient Health Questionnaire - Panic Disorder Module.

**Supplementary Reference**

Brunet, J. P., Tamayo, P., Golub, T. R., & Mesirov, J. P. (2004). Metagenes and molecular pattern discovery using matrix factorization. *Proc Natl Acad Sci U S A, 101*(12), 4164-4169. doi:10.1073/pnas.0308531101

Chen, F., Ke, J., Qi, R., Xu, Q., Zhong, Y., Liu, T., . . . Lu, G. (2018). Increased Inhibition of the Amygdala by the mPFC may Reflect a Resilience Factor in Post-traumatic Stress Disorder: A Resting-State fMRI Granger Causality Analysis. *Front Psychiatry, 9*, 516. doi:10.3389/fpsyt.2018.00516

Chen, H. J., Qi, R., Ke, J., Qiu, J., Xu, Q., Zhang, Z., . . . Chen, F. (2021). Altered dynamic parahippocampus functional connectivity in patients with post-traumatic stress disorder. *World J Biol Psychiatry, 22*(3), 236-245. doi:10.1080/15622975.2020.1785006

Chen, H. J., Zhang, L., Ke, J., Qi, R., Xu, Q., Zhong, Y., . . . Chen, F. (2019). Altered resting-state dorsal anterior cingulate cortex functional connectivity in patients with post-traumatic stress disorder. *Aust N Z J Psychiatry, 53*(1), 68-79. doi:10.1177/0004867418812674

Dai, Y., Zhou, Z., Chen, F., Zhang, L., Ke, J., Qi, R., . . . Zhong, Y. (2023). Altered dynamic functional connectivity associates with post-traumatic stress disorder. *Brain Imaging Behav, 17*(3), 294-305. doi:10.1007/s11682-023-00760-y

Desikan, R. S., Ségonne, F., Fischl, B., Quinn, B. T., Dickerson, B. C., Blacker, D., . . . Killiany, R. J. (2006). An automated labeling system for subdividing the human cerebral cortex on MRI scans into gyral based regions of interest. *NeuroImage, 31*(3), 968-980. doi:10.1016/j.neuroimage.2006.01.021

Ding, H., Zhong, Y., Liu, N., Wu, H., Xu, H., Wu, Y., . . . Wang, C. (2022). Panic disorder aging characteristics: The role of telomerase reverse transcriptase gene and brain function. *Front Aging Neurosci, 14*, 835963. doi:10.3389/fnagi.2022.835963

Fortin, J.-P., Cullen, N., Sheline, Y. I., Taylor, W. D., Aselcioglu, I., Cook, P. A., . . . Shinohara, R. T. (2018). Harmonization of cortical thickness measurements across scanners and sites. *NeuroImage, 167*, 104-120. doi:<https://doi.org/10.1016/j.neuroimage.2017.11.024>

Frigyesi, A., & Höglund, M. (2008). Non-negative matrix factorization for the analysis of complex gene expression data: identification of clinically relevant tumor subtypes. *Cancer Inform, 6*, 275-292. doi:10.4137/cin.s606

Han, X., Jovicich, J., Salat, D., van der Kouwe, A., Quinn, B., Czanner, S., . . . Fischl, B. (2006). Reliability of MRI-derived measurements of human cerebral cortical thickness: the effects of field strength, scanner upgrade and manufacturer. *NeuroImage, 32*(1), 180-194. doi:10.1016/j.neuroimage.2006.02.051

Hao, Z. Y., Zhong, Y., Ma, Z. J., Xu, H. Z., Kong, J. Y., Wu, Z., . . . Wang, C. (2020). Abnormal resting-state functional connectivity of hippocampal subfields in patients with major depressive disorder. *BMC Psychiatry, 20*(1), 71. doi:10.1186/s12888-020-02490-7

Johnson, W. E., Li, C., & Rabinovic, A. (2006). Adjusting batch effects in microarray expression data using empirical Bayes methods. *Biostatistics, 8*(1), 118-127. doi:10.1093/biostatistics/kxj037

Ke, J., Zhang, L., Qi, R., Xu, Q., Zhong, Y., Liu, T., . . . Chen, F. (2018). Typhoon-Related Post-Traumatic Stress Disorder and Trauma Might Lead to Functional Integration Abnormalities in Intra- and Inter-Resting State Networks: a Resting-State Fmri Independent Component Analysis. *Cell Physiol Biochem, 48*(1), 99-110. doi:10.1159/000491666

Lee, D. D., & Seung, H. S. (1999). Learning the parts of objects by non-negative matrix factorization. *Nature, 401*(6755), 788-791. doi:10.1038/44565

Li, Y., Wang, C., Teng, C., Jiao, K., Song, X., Tan, Y., . . . Zhong, Y. (2019). Hippocampus-driving progressive structural alterations in medication-naïve major depressive disorder. *J Affect Disord, 256*, 148-155. doi:10.1016/j.jad.2019.05.053

Liu, T., Ke, J., Qi, R., Zhang, L., Zhang, Z., Xu, Q., . . . Chen, F. (2021). Altered functional connectivity of the amygdala and its subregions in typhoon-related post-traumatic stress disorder. *Brain Behav, 11*(1), e01952. doi:10.1002/brb3.1952

Lovmar, L., Ahlford, A., Jonsson, M., & Syvänen, A. C. (2005). Silhouette scores for assessment of SNP genotype clusters. *BMC Genomics, 6*, 35. doi:10.1186/1471-2164-6-35

Ma, Z., Wang, C., Hines, C. S., Lu, X., Wu, Y., Xu, H., . . . Zhang, N. (2019). Frontoparietal network abnormalities of gray matter volume and functional connectivity in patients with generalized anxiety disorder. *Psychiatry Res Neuroimaging, 286*, 24-30. doi:10.1016/j.pscychresns.2019.03.001

Ma, Z., Zhong, Y., Hines, C. S., Wu, Y., Li, Y., Pang, M., . . . Wang, C. (2020). Identifying generalized anxiety disorder using resting state habenular circuitry. *Brain Imaging Behav, 14*(5), 1406-1418. doi:10.1007/s11682-019-00055-1

Pang, M., Zhong, Y., Hao, Z., Xu, H., Wu, Y., Teng, C., . . . Wang, C. (2021). Resting-state causal connectivity of the bed nucleus of the stria terminalis in panic disorder. *Brain Imaging Behav, 15*(1), 25-35. doi:10.1007/s11682-019-00229-x

Pascual-Montano, A., Carazo, J. M., Kochi, K., Lehmann, D., & Pascual-Marqui, R. D. (2006). Nonsmooth nonnegative matrix factorization (nsNMF). *IEEE Trans Pattern Anal Mach Intell, 28*(3), 403-415. doi:10.1109/tpami.2006.60

Teng, C., Liu, T., Zhang, N., Zhong, Y., & Wang, C. (2022). Cognitive behavioral therapy may rehabilitate abnormally functional communication pattern among the triple-network in major depressive disorder: A follow-up study. *J Affect Disord, 304*, 28-39. doi:10.1016/j.jad.2022.02.050

Weng, Y., Qi, R., Chen, F., Ke, J., Xu, Q., Zhong, Y., . . . Lu, G. (2018). The Temporal Propagation of Intrinsic Brain Activity Associate With the Occurrence of PTSD. *Front Psychiatry, 9*, 218. doi:10.3389/fpsyt.2018.00218

Wu, H., Zhong, Y., Xu, H., Ding, H., Yuan, S., Wu, Y., . . . Wang, C. (2022). Glutamic Acid Decarboxylase 1 Gene Methylation and Panic Disorder Severity: Making the Connection by Brain Gray Matter Volume. *Front Psychiatry, 13*, 853613. doi:10.3389/fpsyt.2022.853613

Wu, Y., Zhong, Y., Zheng, G., Liu, Y., Pang, M., Xu, H., . . . Zhang, N. (2022). Disrupted fronto-temporal function in panic disorder: a resting-state connectome study. *Brain Imaging Behav, 16*(2), 888-898. doi:10.1007/s11682-021-00563-z

Wu, Z., Wang, C., Dai, Y., Xiao, C., Zhang, N., & Zhong, Y. (2022). The effect of early cognitive behavior therapy for first-episode treatment-naive major depressive disorder. *J Affect Disord, 308*, 31-38. doi:10.1016/j.jad.2022.04.008

Wu, Z., Wang, C., Ma, Z., Pang, M., Wu, Y., Zhang, N., & Zhong, Y. (2020). Abnormal functional connectivity of habenula in untreated patients with first-episode major depressive disorder. *Psychiatry Res, 285*, 112837. doi:10.1016/j.psychres.2020.112837

Xiao, Y., Chen, F., Lei, W., Ke, J., Dai, Y., Qi, R., . . . Zhong, Y. (2023). Transcriptional signal and cell specificity of genes related to cortical structural differences of post-traumatic stress disorder. *J Psychiatr Res, 160*, 28-37. doi:10.1016/j.jpsychires.2023.02.002

Xu, H., Zhong, Y., Yuan, S., Wu, Y., Ma, Z., Hao, Z., . . . Zhang, N. (2021). Nitric Oxide Synthase Type 1 Methylation Is Associated With White Matter Microstructure in the Corpus Callosum and Greater Panic Disorder Severity Among Panic Disorder Patients. *Front Neurol, 12*, 755270. doi:10.3389/fneur.2021.755270

Xu, L., Xu, H., Ding, H., Li, J., & Wang, C. (2021). Intrinsic Network Brain Dysfunction Correlates With Temporal Complexity in Generalized Anxiety Disorder and Panic Disorder. *Front Hum Neurosci, 15*, 647518. doi:10.3389/fnhum.2021.647518

Xu, T., Chen, F., Zhang, L., Dai, Y., Ke, J., Qi, R., . . . Zhong, Y. (2022). Study on the changes of Structural Covariance Network in post-traumatic stress disorder. *Brain Imaging Behav, 16*(5), 1992-2000. doi:10.1007/s11682-022-00669-y

Yan, C. G., Wang, X. D., Zuo, X. N., & Zang, Y. F. (2016). DPABI: Data Processing & Analysis for (Resting-State) Brain Imaging. *Neuroinformatics, 14*(3), 339-351. doi:10.1007/s12021-016-9299-4

Yu, Q., Wang, C., Xu, H., Wu, Y., Ding, H., Liu, N., . . . Wang, C. (2022). The mediating role of transmembrane protein 132D methylation in predicting the occurrence of panic disorder in physical abuse. *Front Psychiatry, 13*, 972522. doi:10.3389/fpsyt.2022.972522

Zhang, D., Teng, C., Xu, Y., Tian, L., Cao, P., Wang, X., . . . Hu, X. (2024). Genetic and molecular correlates of cortical thickness alterations in adults with obsessive-compulsive disorder: a transcription-neuroimaging association analysis - CORRIGENDUM. *Psychol Med, 54*(14), 1. doi:10.1017/s003329172400271x

Zhou, W., Yuan, Z., Yingliang, D., Chaoyong, X., Ning, Z., & Chun, W. (2021). Differential patterns of dynamic functional connectivity variability in major depressive disorder treated with cognitive behavioral therapy. *J Affect Disord, 291*, 322-328. doi:10.1016/j.jad.2021.05.017
